# Lung Cancer Screening and USPSTF Recommendations

**DOI:** 10.1001/jamanetworkopen.2024.58916

**Published:** 2025-02-10

**Authors:** Michael E. Darden, Alex Hoagland

**Affiliations:** 1 Johns Hopkins University Carey Business School, Washington, DC; 2 National Bureau of Economic Research, Cambridge, Massachusetts; 3 Institute of Health Policy, Management, and Evaluation, University of Toronto, Toronto, Ontario, Canada

## Abstract

This cross-sectional study examines the prevalence of lung cancer screening outside USPSTF (US Preventive Services Task Force) recommendations among individuals with smoking history.

## Introduction

Lung cancer remains the leading cause of death from cancer.^1^ For persons at high risk, low-dose computed topography screening effectively identifies early-stage lung cancer and can reduce lung cancer mortality.^2^ In 2021, the US Preventive Services Task Force (USPSTF) broadened recommended lung cancer screening (LCS) guidelines to include adults aged 50 to 79 years with a cigarette smoking history of 20 or more years who are current or recent (within 15 years) smokers.^3^ Studies of LCS prevalence have examined data from either the population who meet all USPSTF criteria^4^ or selected LCS registries.^5^ In other contexts, such as breast cancer, screening outside USPSTF recommendations is common, and those choosing screening outside recommendations are often at considerably higher risk.^6^ Using nationally representative data, we investigated the prevalence of LCS by eligibility with respect to USPSTF criteria, and we compared those choosing screening by eligibility.

## Methods

This cross-sectional study analyzed Behavioral Risk Factor Surveillance System (BRFSS) data from 2022. We focused on respondents with some smoking history (pack-years *>*0) and nonmissing information on LCS, smoking history, and age. We examined how rates of past year LCS changed at thresholds for USPSTF guidelines. Specifically, we estimated the fraction of those undergoing screening stratified by their screening recommendation status around thresholds for age and pack-years. We also compared demographic, socioeconomic, and health characteristics among those undergoing screening for lung cancer in the last year by whether the respondent met USPSTF recommendation guidelines. We calculated *P* values for differences in means across groups using standard 2-sided *t* tests. Statistical significance was assessed at the *P* = .05 level. In all analyses, we used BRFSS survey weights. All analyses were conducted using Stata 15.1 software (StataCorp LLC). Where appropriate, analysis followed STROBE reporting guidelines. This study was based on deidentified publicly available information and exempt from approval by the Johns Hopkins University Homewood Institutional Review Board.

## Results

Of the sample of 108 421 respondents, 7.0% (95% CI, 6.7%-7.3%) claimed to have undergone LCS in the last year. Of these respondents, 54.8% (95% CI, 52.4%-57.1%) met all USPSTF recommendation criteria. While 75.7% (95% CI, 72.1%-79.2%) of ineligible screeners met the age criterion (≥50 years), only 26.6% (95% CI, 23.4%-29.8%) had a smoking history of 20 or more pack-years (Table). Panel A of the Figure shows screening prevalence by age for those otherwise recommended to undergo screening and those not meeting the pack-year criteria. Panel B of the Figure shows screening prevalence by pack-years for those otherwise recommended (ie, aged ≥50 years) and those younger than 50 years.

**Table.** Sample Characteristics by Past Year Screening and USPSTF Recommendations^a^

Recommendation criteria	Weighted mean proportion, %				*P* value
	Full sample (N = 108 421)	Screening (n = 8389 [7.0%])			
		All	USPSTF recommended^b^		
			Yes (54.8%)	No (45.2%)	
Age, y	53.7	63.2	65.8	60.0	<.001
Pack-years	19.7	35.4	49.7	18.1	<.001
Current smoker	38.5	47.4	56.3	36.6	<.001
Time cessation if former smoker, y	19.1	16.0	7.2	23.4	<.001
Health characteristics					
Health insurance	91.8	97.8	98.9	96.4	.01
Subjective health					
Excellent, very good, or good	75.5	57.9	54.8	61.7	.003
Fair or poor	24.5	42.1	45.2	38.3	
Poor physical health, No. of d per mo					
0	57.0	44.0	43.0	45.1	.41
1-13	24.6	25.3	25.0	25.6	.80
≥14	18.4	30.8	32.0	29.3	.26
Poor mental health, No. d per mo					
0	54.9	56.5	58.5	54.0	.07
1-13	25.2	22.5	20.8	24.6	.06
≥14	19.9	21.0	20.7	21.4	.77
BMI *>*30	37.9	35.7	33.6	38.2	.06
History of COVID-19	32.1	26.5	22.2	31.8	<.001
History of lung disease^c^	14.2	44.0	55.4	30.2	<.001
Demographic and SES characteristics					
Sex					
Male	54.3	54.8	55.2	54.4	.74
Female	45.7	45.2	44.8	45.6	.74
Race and ethnicity					
Asian	3.0	1.6	1.8	1.3	.64
Black	9.5	12.3	8.7	16.6	<.001
Hispanic	12.6	8.9	5.9	12.5	<.001
White	69.3	72.3	79.7	63.3	<.001
Other^d^	5.6	5.0	3.9	6.3	.06
Education					
*<*High school	14.0	17.5	15.4	20.0	.05
High school	29.8	32.8	35.9	29.0	<.001
Some college	34.2	34.7	35.6	33.6	.38
≥College	21.9	15.1	13.1	17.4	<.001
Metro residence	82.1	82.0	80.0	84.5	<.001
Employed^e^	55.4	29.3	23.7	36.2	<.001

Abbreviations: BMI, body mass index (calculated as weight in kilograms divided by height in meters squared); SES, socioeconomic status; USPSTF, US Preventive Services Task Force.

^a^ Sample data from the 2022 Behavioral Risk Factor Surveillance System.

^b^ USPSTF recommended screening: 20 or more pack-years, aged 50 or more years, and current or recent smoker.

^c^ Lung disease includes chronic obstructive pulmonary disease, emphysema, and chronic bronchitis.

^d^ Other race includes Native American, Pacific Islander, and multiple races.

^e^ Employed includes self-employed.

**Figure.**
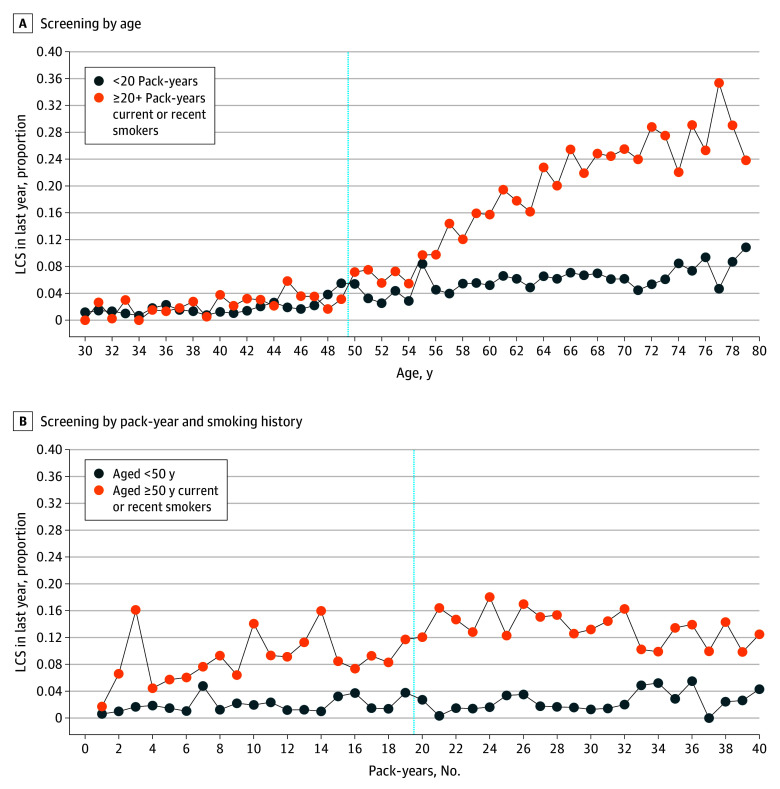
Age and Pack-Year Discontinuity A, For those represented on the current or recent smokers line, age threshold of 50 years pushes them into recommended status. B, For those represented on the current or recent smokers line, 20 pack-year threshold pushes them into recommended status. LCS indicates lung cancer screening.

Relative to eligible screeners, ineligible screeners were less likely to have health insurance (96.4% vs 98.9%; *P* = .01), but they were more likely to be in excellent/very good/good subjective health (61.7% vs 54.8%; *P* < .001) (Table). Ineligible screeners were significantly less likely to have a history of lung disease (30.2% vs 55.4%; *P* < .001) but more likely to have a history of COVID-19 (31.8% vs 22.2%; *P* < .001), and they were less likely to be White (63.3% vs 79.7%; *P* < .001), more likely to have less than a high school education (20.0% vs 15.4%; *P* = .05), and more likely to have a college education (17.4% vs 13.1%; *P* < .001).

## Discussion

We found that nearly half of all lung cancer screening occurred outside USPSTF recommendations, based on older respondents (≥50 years) with some smoking history (1≤pack-years <20). We found that those ineligible screeners had better subjective health, which raises the question of why these individuals seek screening. One theory is that older former smokers recognize that most lung cancer occurs after age 65 years, and they worry about their risk. The main limitation of our study was that lung cancer and smoking information was based on self-reports. Future research should investigate the benefit of LCS outside USPSTF recommendations vs the risks.

## Notes

Supplement.
Data Sharing Statement
