# Supplementary material for: Lung Cancer Screening and USPSTF Recommendations
Source: JAMA Netw Open. 2025 Feb 10;8(2):e2458916. doi: 10.1001/jamanetworkopen.2024.58916 (PMC11811788; doi:10.1001/jamanetworkopen.2024.58916)
Supplement: Supplement. — Data Sharing Statement [file jamanetwopen-e2458916-s001.pdf]

Data Sharing Statement

Darden. Lung Cancer Screening and USPSTF Recommendations. *JAMA Netw Open*.
Published February 10, 2025. doi:10.1001/jamanetworkopen.2024.58916

Data
Data available: Yes
Data types: Deidentified participant data
How to access data: Data and Code will be available upon request at mdarden4@jhu.edu
When available: With publication

Supporting Documents
Document types: Statistical/analytic code
How to access documents: mdarden4@jhu.edu
When available: With publication

Additional Information
Who can access the data: Anyone requesting the data.
Types of analyses: Replication
Mechanisms of data availability: Without investigator support.
Any additional restrictions: None
